# TAF4b Regulates Oocyte-Specific Genes Essential for Meiosis

**DOI:** 10.1371/journal.pgen.1006128

**Published:** 2016-06-24

**Authors:** Kathryn J. Grive, Eric A. Gustafson, Kimberly A. Seymour, Melody Baddoo, Christoph Schorl, Kayla Golnoski, Aleksandar Rajkovic, Alexander S. Brodsky, Richard N. Freiman

**Affiliations:** 1 MCB Graduate Program, Brown University, Providence, Rhode Island, United States of America; 2 MCB Department, Brown University, Providence, Rhode Island, United States of America; 3 School of Medicine, Tulane University, New Orleans, Louisiana, United States of America; 4 Magee Women’s Research Institute, Pittsburgh, Pennsylvania, United States of America; 5 Department of Pathology, Rhode Island Hospital and Brown University, Providence, Rhode Island, United States of America; Stanford University School of Medicine, UNITED STATES

## Abstract

TAF4b is a gonadal-enriched subunit of the general transcription factor TFIID that is implicated in promoting healthy ovarian aging and female fertility in mice and humans. To further explore the potential mechanism of TAF4b in promoting ovarian follicle development, we analyzed global gene expression at multiple time points in the human fetal ovary. This computational analysis revealed coordinate expression of human *TAF4B* and critical regulators and effectors of meiosis I including *SYCP3*, *YBX2*, *STAG3*, and *DAZL*. To address the functional relevance of this analysis, we turned to the embryonic *Taf4b*-deficient mouse ovary where, for the first time, we demonstrate, severe deficits in prophase I progression as well as asynapsis in *Taf4b*-deficient oocytes. Accordingly, TAF4b occupies the proximal promoters of many essential meiosis and oogenesis regulators, including *Stra8*, *Dazl*, *Figla*, and *Nobox*, and is required for their proper expression. These data reveal a novel TAF4b function in regulating a meiotic gene expression program in early mouse oogenesis, and support the existence of a highly conserved TAF4b-dependent gene regulatory network promoting early oocyte development in both mice and women.

## Author Summary

Proper regulation of early oogenesis is essential for long-term ovarian health and fertility, as female mammals (and women) possess a finite pool of oocytes at birth. Meiotic progression during these early stages of oogenesis ensures genomic integrity and proper chromosome segregation in the reproductive years and decades to come. We investigated the role of transcription factor TAF4b in proper expression of meiosis genes and in the proper progression through prophase I. We have identified a novel function for TAF4b in promoting appropriate expression of critical meiosis genes including *Stra8*, *Sycp1*, *Sycp2*, and *Msy2*. Furthermore, we have demonstrated TAF4b occupancy at the proximal promoters of *Figla*, *Nobox*, and *Dazl*. This occupancy is crucial for the chromosomal events of prophase I, as *Taf4b*-deficent oocytes experience defects in meiosis I, a high incidence of asynapsis, and disrupted recombination. These data identify TAF4b as a novel upstream transcriptional regulator of the early meiotic program that is essential for healthy oogenesis.

## Introduction

One of the most fundamental aspects of germline regulation is meiotic progression, in which germ cell chromatin acquires a new configuration [1], genetic material is exchanged between homologous chromosomes, and germ cells are ultimately reduced to half the genetic material of the parental cell [2]. In mice, oogonia enter into meiosis I at embryonic day 13.5 (E13.5) after retinoic acid (RA) production activates the Stimulated by Retinoic Acid 8 (*Stra8*) gene [3–6]. Oocytes progress through the stages of prophase I of meiosis I with the acquisition of meiotic double strand breaks in leptotene, assembly of the synaptonemal complex in zygotene, completion of synapsis by pachytene, and finally arrest in diplotene, after the resolution of meiotic recombination [7,8]. Meiotic onset also occurs in human oocytes within the fetal human ovary around gestational weeks 11–12, after which diplotene arrest begins around 16–20 weeks [8–10]. Remarkably, these arrested oocytes remain in diplotene until the first meiotic division just prior to ovulation [7], which will not occur until months later in mice and decades later in humans. In women, these oocytes may not be utilized for conception until four to five decades after their initial formation and arrest. Therefore, these early embryonic meiotic events must not only take place with great fidelity, but must ensure long-term genomic integrity to confer proper fertilization and development.

Intricate regulation of gene expression is critical for both the meiotic program and the subsequent packaging of meiotically arrested oocytes into primordial follicles. A number of transcription factors are known to play crucial roles in these processes as well as in germ cell cyst breakdown including Factor in the Germline Alpha (FIGLα) [11,12] and Newborn Ovary Homeobox Gene (NOBOX) [13–15]. Another promising candidate for meiosis and ovarian reserve regulation is TAF4b, a gonadal-enriched subunit of the TFIID complex that is critical for female fertility in the mouse [16]. TFIID is a multi-protein general transcription factor complex composed of the TATA-box binding protein (TBP) and 14 TBP-associated factors (TAFs). *Taf4b*-deficient female mice suffer from hallmarks of primary ovarian insufficiency (POI) including persistent estrous, elevated serum follicle stimulating hormone (FSH) [17] and accelerated primordial follicle depletion [18]. We have recently shown that TAF4b is required for proper germ cell cyst breakdown, primordial follicle assembly and oocyte survival during the window of meiotic progression in the perinatal mouse ovary [19]. In the context of human oocyte preservation, a report by the Dutch premature ovarian failure (POF) Consortium linked single nucleotide polymorphism (SNP) variation in the human *TAF4B* gene to POI [20], while a report of human oocyte quality has reported *TAF4B* expression as a positive correlate of increased oocyte quality [21]. Even though the crucial developmental functions of TAF4b in the developing murine ovarian reserve have been established, the precise functions of TAF4b in the early oocyte and its potential mechanisms of oocyte-specific gene regulation remain poorly understood.

To get a better understanding of TAF4B’s potential roles in human oocyte development, we utilized a data set profiling global gene expression in the human fetal ovary [22]. From our analysis, we found that human *TAF4B* expression is highly correlated with the expression of critical meiotic regulators including *SYCP3*, Y Box Binding Protein 2 (*YBX2*, also known as *MSY2)*, and Deleted in Azoospermia-Like (*DAZL*). Furthermore, to elucidate fundamental molecular mechanisms associated with healthy meiotic progression and ovarian reserve establishment, we have analyzed prophase I events in the context of *Taf4b*-deficient ovaries at E16.5. Importantly, while *Taf4b*-deficient ovaries experience accelerated germ cell death immediately after birth, they possess normal germ cell densities during late embryogenesis, allowing us to compare these cell populations [19]. The present analysis of meiosis I in embryonic *Taf4b*-deficient ovaries revealed altered prophase I progression and abundant asynapsis as well as altered diplotene arrest in *Taf4b*-deficient oocytes. We have also observed deficits in the ability of *Taf4b*-deficient oocytes to perform meiotic homologous recombination. Finally, chromatin immunoprecipitation (ChIP) assays demonstrated direct occupancy of TAF4b at the proximal promoter regions of critical meiotic and oogenesis regulators including *Stra8*, *Dazl*, *Figla*, and *Nobox*, placing TAF4b upstream of these essential regulators of oogenesis. Together, these data help explain the critical role of TAF4b in the establishment of the postnatal primordial follicle pool, and identify novel functions for TAF4b in the orchestration of early meiotic events.

## Results

### Coordinate expression of Human *TAF4B* with expression of meiotic regulators and effectors

**Fig 1:**
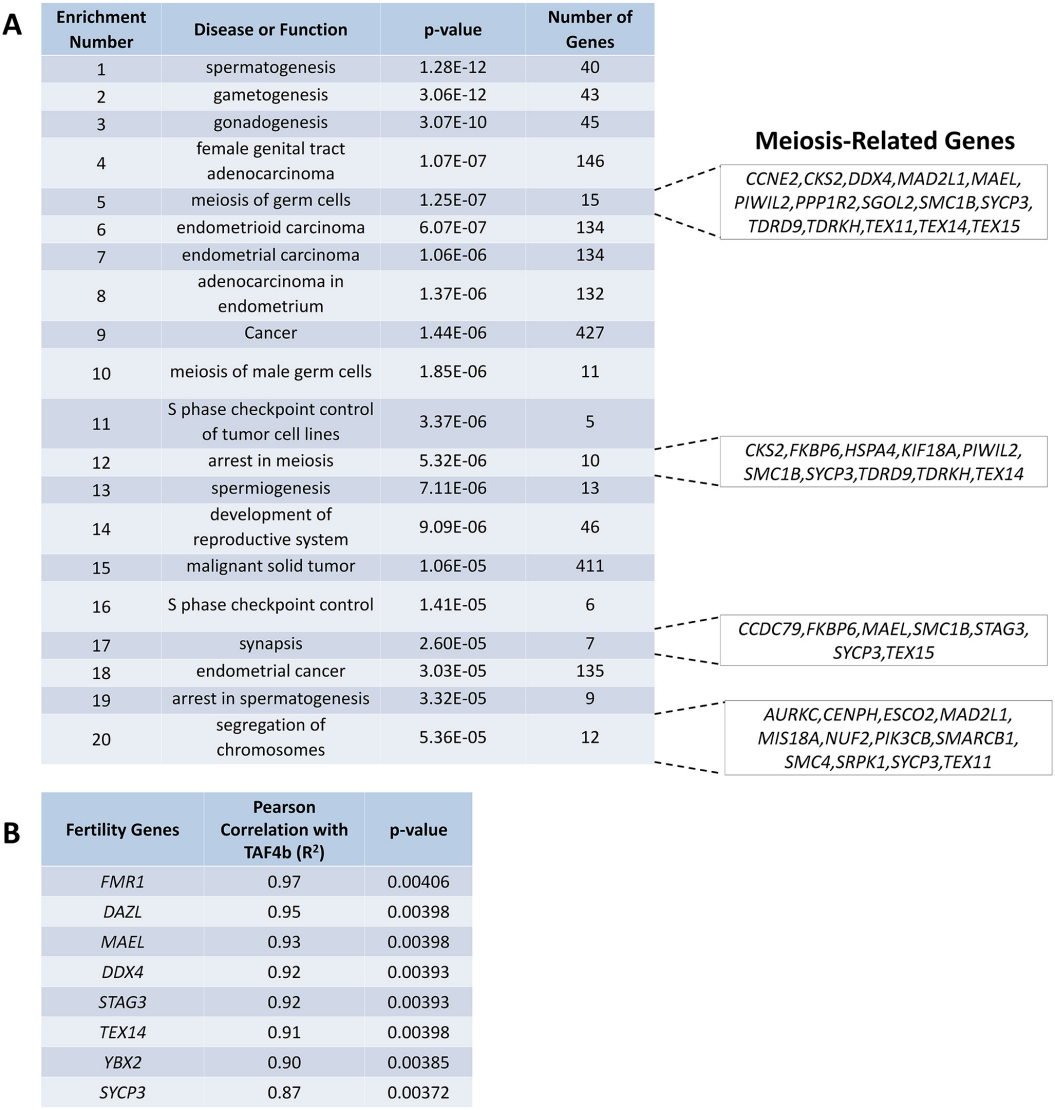
Human *TAF4B* expression is highly correlated with the expression of meiotic regulators. (A) Ingenuity Pathway Analysis was performed on an existing data set profiling gene expression in human fetal ovary to determine coordinate regulation of genes with human *TAF4B*. The top twenty most-significant enrichments from this analysis revealed many functions related to meiotic regulation including *SYCP3*, *STAG3*, *YBX2*, and *DAZL*. (B) Pearson correlations were calculated for fertility genes of interest and R^2^ values displayed.

To gain a better understanding of the potential molecular functions of TAF4B in human oogenesis, we examined coordinate *TAF4B* gene expression profiles in the human fetal ovary over gestational time [22], reasoning that the most essential functions of TAF4B may be highly conserved between mice and humans. We identified the genes that are most correlated with *TAF4B* expression during human ovarian development (S1 Table). To test if the list of genes highly correlated with *TAF4B* is enriched for specific functions, we evaluated the top 624 genes with Pearson correlations >0.85 for enriched pathways. Enrichment determined using Ingenuity Pathway Analysis (IPA) found that *TAF4B* expression is most highly correlated with the expression of a network of meiotic regulators and effectors during human fetal ovarian development (Fig 1A).

Pearson Coefficient values for a number of notable genes implicated in meiosis were calculated (Fig 1B), including *SYCP3* (R^2^ = 0.87, *P*-value = 0.004), *YBX2* (R^2^ = 0.90, *P*-value = 0.004), *DAZL* (R^2^ = 0.95, *P*-value = 0.004), the human ortholog of *Dazl*, *MAEL* (R^2^ = 0.93, *P*-value = 0.00398), a piRNA-pathway regulating gene essential for prophase progression and oocyte survival [23], and *STAG3* (R^2^ = 0.92, *P*-value = 0.00393), a cohesin required for proper synapsis [24–26]. Interestingly, genes involved in primordial follicle formation were also highly correlated including *TEX14* (R^2^ = 0.91, *P*-value = 0.004), a component of intercellular bridges in germ cell cysts [27,28], *DDX4* (R^2^ = 0.92, *P*-value = 0.004), the human ortholog of *Vasa* [29,30], and *FMR1* (R^2^ = 0.97, *P*-value = 0.004), an RNA-binding protein associated with premature ovarian failure [31,32], among others. Together these data suggest that TAF4B may execute a highly conserved role in mammalian meiosis I regulation that is critical for healthy oocyte development and ovarian aging in women.

### *Taf4b*-deficient oocytes experience defective meiotic progression and chromosome synapsis at E16.5

**Fig 2:**
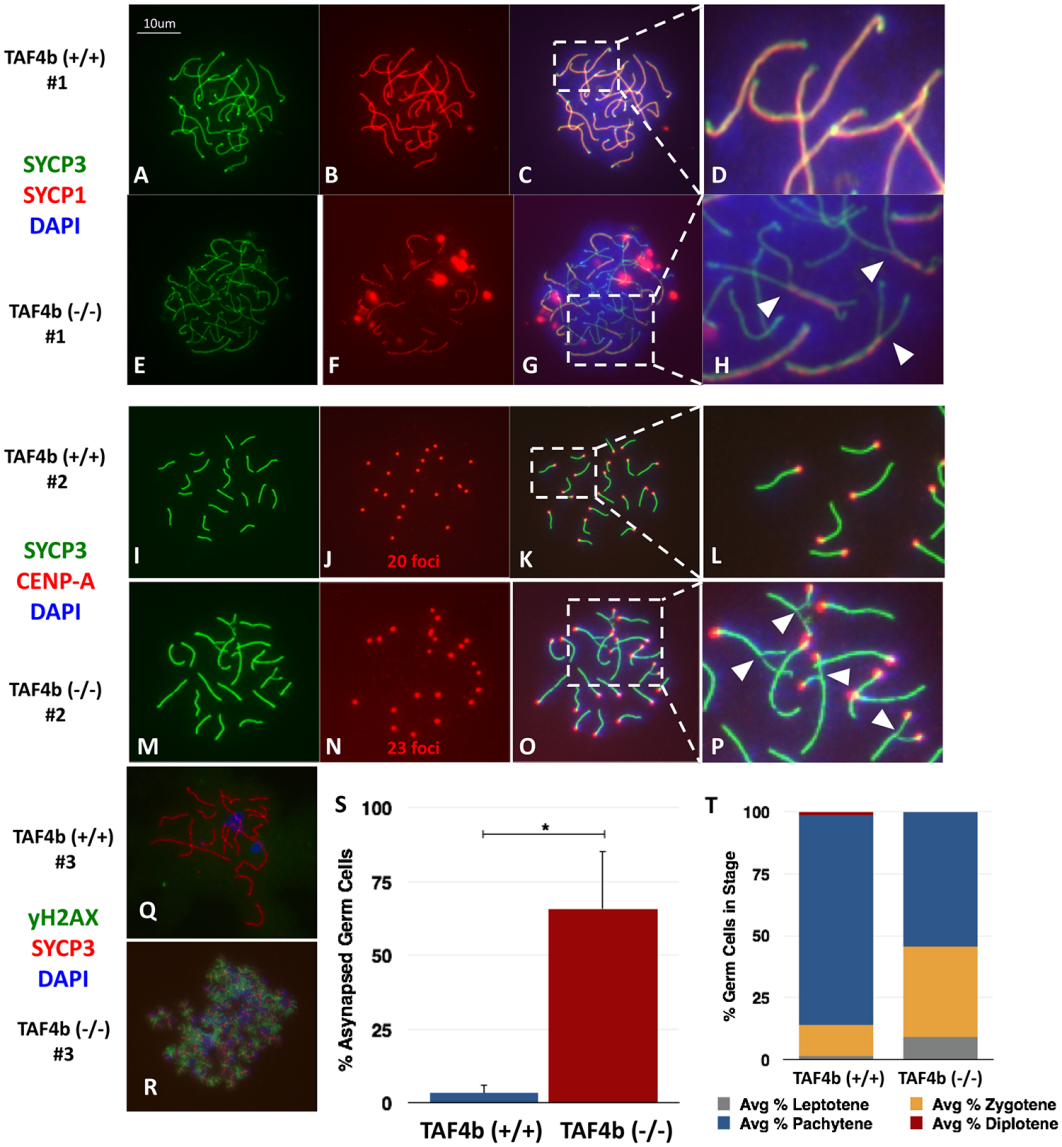
*Taf4b*-deficient oocytes experience defective meiotic progression and chromosome asynapsis. Pachytene spreads were prepared using the drying-down technique (50) on cell suspensions from E16.5 wild-type (A-D; I-L) and *Taf4b*-deficient (E-H; M-P) ovaries. Slides were stained for SYCP1 and SYCP3 (A-H), or SYCP3 and CENP-A (I-P). White arrowheads in (H) indicate regions of asynapsis in which SYCP3 is localized but SYCP1 is not; while white arrowheads in (P) indicate regions of asynapsis, many of which can be visualized by non-overlapping CENP-A centromeric foci. Slides were also stained for γH2AX and SYCP3 (Q, R) to visualize double-strand breaks. A high incidence of asynapsis (S), as scored by >20 CENP-A foci, as well as defects in meiotic progression (T), as scored by SYCP3 configuration (S1 Fig), were apparent in *Taf4b*-deficient oocytes. *: n = 4 animals each, one-tailed t-test, p<0.05

As many genes coordinately expressed with human *TAF4B* in the human fetal ovary are critical for the fidelity of meiosis I, we analyzed prophase I progression in *Taf4b*-deficient mouse oocytes. Strikingly, when visualized by two different immunofluorescence methods, E16.5 *Taf4b*-deficient oocytes exhibit high degrees of asynapsis and aberrant meiosis. One means to test synapsis is by visualizing chromosomal co-localization of Synaptonemal Complex Proteins 1 and 3, which coat the central and lateral elements, respectively [33]. Complete co-localization indicates faithful synapsis, as seen in wild-type oocytes (Fig 2A–2D), while regions of asynapsis only stain positively for SYCP3 and lack SYCP1, as observed in most *Taf4b*-deficient oocytes (Fig 2E–2H). Another means of testing synapsis is by counting chromosomal centromeric foci marked by Centromere Protein A (CENP-A) staining [34]. Complete synapsis of XX pachytene chromosomes will result in the appearance of 20 centromeric foci, as seen in wild-type oocytes (Fig 2I–2L), while regions of asynapsis will result in non-overlapped centromeres and the appearance of greater than 20 CENP-A foci as seen in most *Taf4b*-deficient oocytes (Fig 2M–2P). Persistent double strand breaks are also evident in *Taf4b*-deficient oocytes as visualized by the presence of the histone variant γH2AX on pachytene chromosomes (Fig 2Q and 2R). Notably, nearly 75% of *Taf4b*-deficient oocytes exhibit some degree of asynapsis during pachytene, a percentage significantly greater than that ever observed in wild-type oocytes (Fig 2S, p < 0.05).

**Fig 3:**
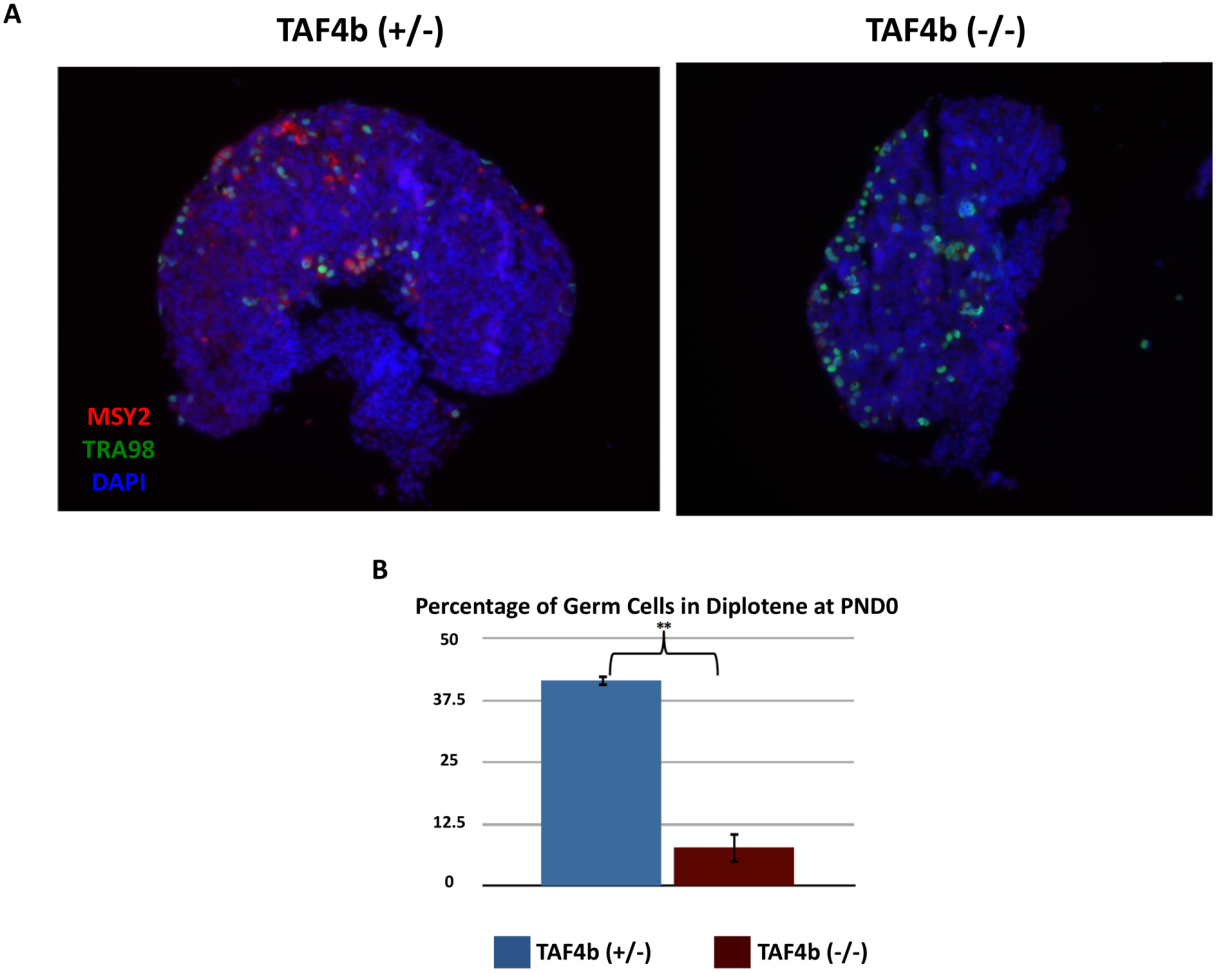
Reduced MSY2 expression and diplotene arrest in *Taf4b*-deficient oocytes. (A) PND0 wild-type and *Taf4b*-deficient ovary tissue sections were stained with primary antibodies against MSY2 and germ cell marker TRA98, which were then quantified for the number of MSY2+/TRA98+ double-positive cells (B). As MSY2 indicates diplotene arrest, the data suggest a defect in diplotene arrival in *Taf4b*-deficient oocytes.

In addition to clear deficits in synapsis during pachytene, *Taf4b*-deficient oocytes also experience a prophase I progression defect. Meiotic stages of oocytes were determined by the configuration of SYCP3 staining at E16.5 (S1 Fig). While nearly 90% of wild-type oocytes have reached pachytene by E16.5, *Taf4b*-deficient oocytes persist in the earlier stages of leptotene and zygotene with only about 50% reaching pachytene at the same time (Fig 2T). Moreover, using MSY2 as a marker of diplotene arrest at PND0, we found that while about 40% of wild-type oocytes (visualized by TRA98 staining) have reached this developmental milestone, fewer than 10% of *Taf4b*-deficient oocytes have reached this arrest (Fig 3). Strikingly, just one day later, at PND1, most *Taf4b*-deficient oocytes undergo apoptotic cell death [19].

**Fig 4:**
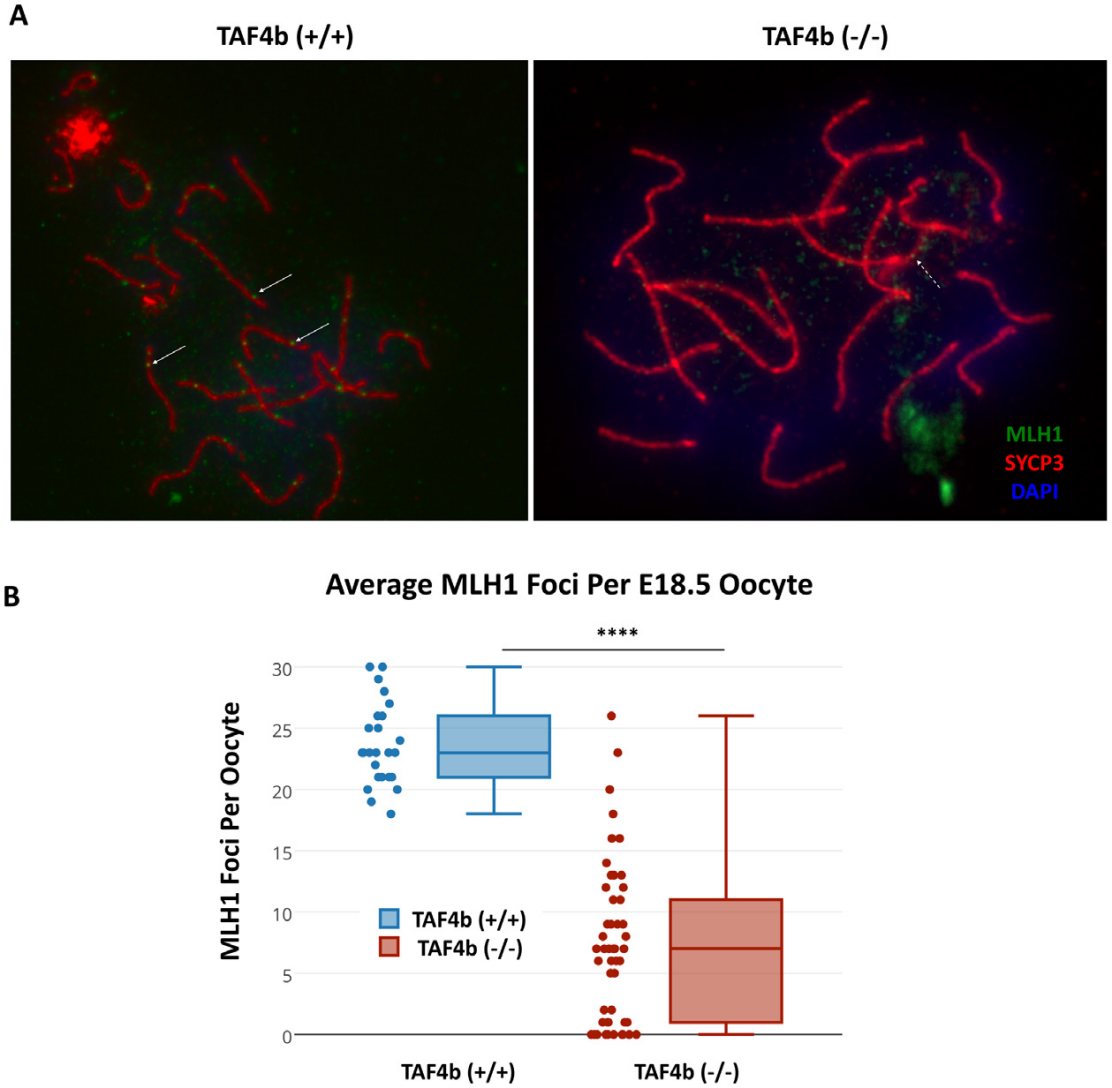
Reduced MLH1 meiotic recombination foci on pachytene chromosomes in *Taf4b*-deficient oocytes. (A) Oocyte meiotic chromosome spreads were prepared from E18.5 wild-type and *Taf4b*-deficient ovaries, and stained with primary antibodies against SYCP3 and MLH1. While wild-type oocytes have one or two intensely-stained MLH1 foci per homologous pair (white solid arrows), few of these foci are evident in *Taf4b*-deficient oocytes. Instead, the majority of MLH1 foci visible in *Taf4b*-deficient oocytes are comparatively faint (white dashed arrows) (B) Quantification of average MLH1 foci per oocyte. *Taf4b*-deficient oocytes possess significantly fewer total MLH1 foci than wild-type oocytes. *: n = 3 animals with 25 or more pachytene oocytes each, one-tailed t-test, p<0.0001

Finally, as proper synapsis is known to initiate at sites of recombination, the fidelity of meiotic recombination was tested by visualizing MLH1 foci on pachytene chromosomes. While wild-type oocytes possess one or two strongly-stained foci per homologous pair, *Taf4b*-deficient oocytes lack many of these sites as well as an overall decreased intensity of staining (Fig 4A). Quantification revealed a significant reduction of overall number of MLH1 foci on chromosomes from *Taf4b*-deficient oocytes (p<0.0001) (Fig 4B). Wild-type oocytes average about 23 foci per cell, while *Taf4b*-deficient oocytes average about 8 foci per cell with increased variability including many oocytes with no quantifiable foci. Given the loss of *Taf4b*-deficient oocytes just days after these phenotypes are observed, the data suggest a TAF4b-dependent link between the ability to correctly progress and arrest in prophase I during embryogenesis and postnatal oocyte survival.

### TAF4b directly occupies promoter regions of critical meiosis and oogenesis regulators

**Fig 5:**
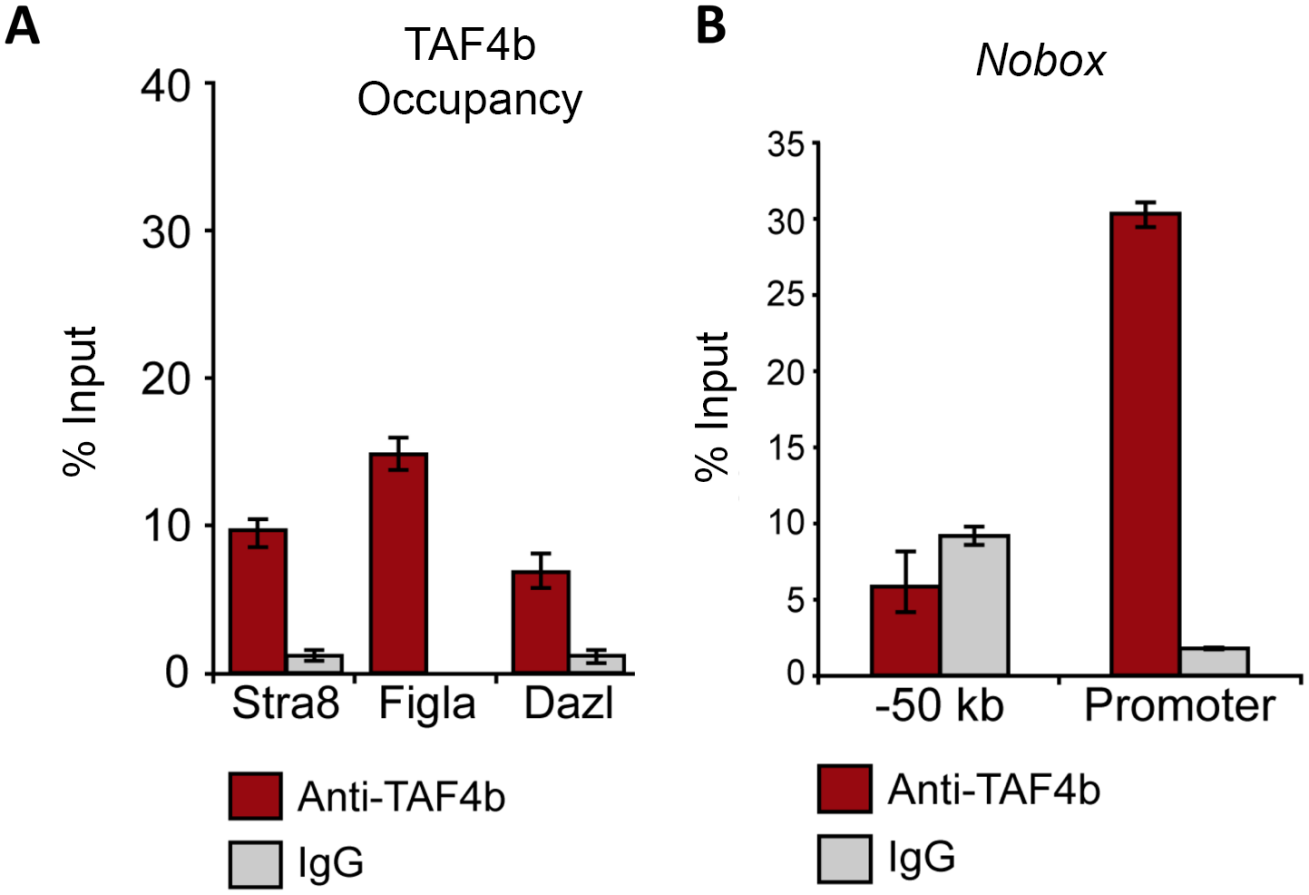
TAF4b targets the promoters of critical meiosis and oogenesis regulators. (A) Wild-type E18.5 ovarian chromatin pulled down by antibodies against TAF4b or IgG and then qPCR-amplified using primers against the proximal promoters of *Stra8*, *Dazl*, *Figlα*, *Nobox* and a non-genic region upstream of *Nobox*. qPCR demonstrates enriched recovery of these proximal promoters with TAF4b-precipitated chromatin relative to IgG. (B) The *Nobox* proximal promoter, in particular, demonstrates enrichment for TAF4b, in contrast to a non-genic region upstream of the promoter. For all analyses, data from each primer set were normalized to the E18.5 mouse ovary genomic DNA input levels and represented as a percentage of that DNA input. Each qPCR reaction was performed in triplicate and averaged. Error bars indicate the normalized standard deviation resulting from experimental triplicate qPCR reactions.

While TAF4b is evidently required for proper regulation of meiosis I progression, the nature of this regulation was unclear. To test if TAF4b directly occupies the proximal promoters of essential meiosis genes, we performed ChIP from wild-type E18.5 ovaries using a validated anti-TAF4b antibody (S2 Fig) or a negative control IgG antibody, and performed quantitative PCR. Strikingly, immunoprecipitated chromatin bound by TAF4b included the proximal promoters of *Stra8* and *Dazl* (Fig 5A). As oogenesis regulators *Figlα* and *Nobox* are known downstream targets of DAZL [35], these promoters were also tested and found to be directly bound by TAF4b (Fig 5A and 5B). TAF4b occupancy at these important loci is specific, as genomic regions not expected to be occupied by TAF4b, including a non-genic region 50kb upstream of *Nobox* were not enriched for TAF4b (Fig 5B). Quantitative PCR results were validated by gel electrophoresis and visualization of amplified proximal promoters (S3 Fig).

**Fig 6:**
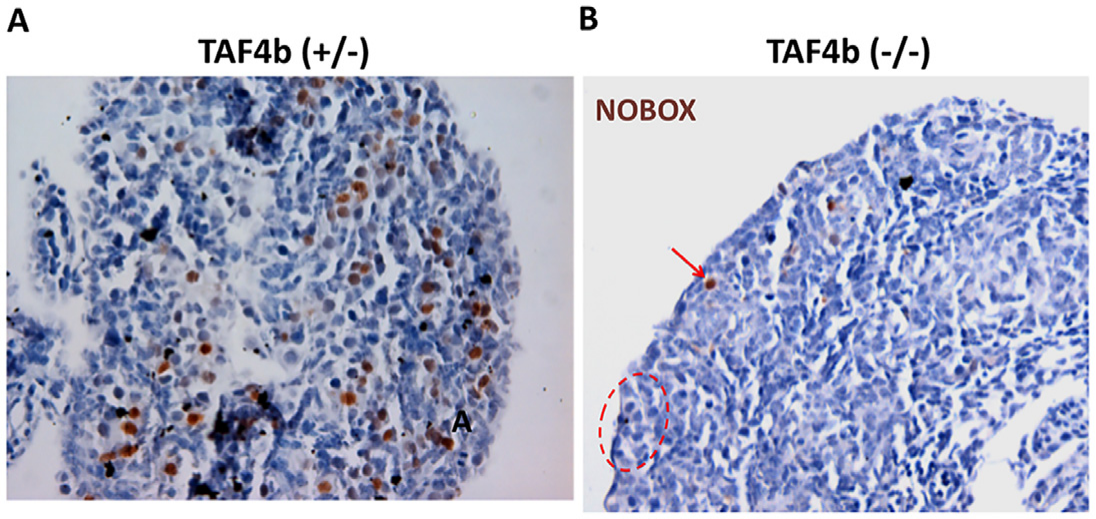
Reduced NOBOX protein expression in *Taf4b*-deficient ovaries. (A) Immunohistochemistry detecting NOBOX protein was performed on E18.5 *Taf4b*-heterozygous and (B) *Taf4b*–deficient ovary tissue sections. While some *Taf4b*-deficient oocytes still express high levels of NOBOX (red arrow), many germ cells possess little to no NOBOX expression (dashed red circle).

To determine whether TAF4b occupancy at some of these promoters correlates with differences in their protein expression, E18.5 *Taf4b*-heterozygous and *Taf4b*-deficient ovaries were stained by immunohistochemistry for NOBOX protein. Although there are no statistically significant differences in germ cell density at this time point [19], NOBOX expression is dramatically reduced in *Taf4b*-deficient oocytes (Fig 6A and 6B). Additionally, PND0 *Taf4b*-heterozygous and *Taf4b*-deficient ovaries were stained by immunofluorescence for DAZL protein, which was also observed to be dramatically reduced in *Taf4b*-deficient oocytes (S4A and S4B Fig), a result which was confirmed by immunoblotting (S4C Fig).

**Fig 7:**
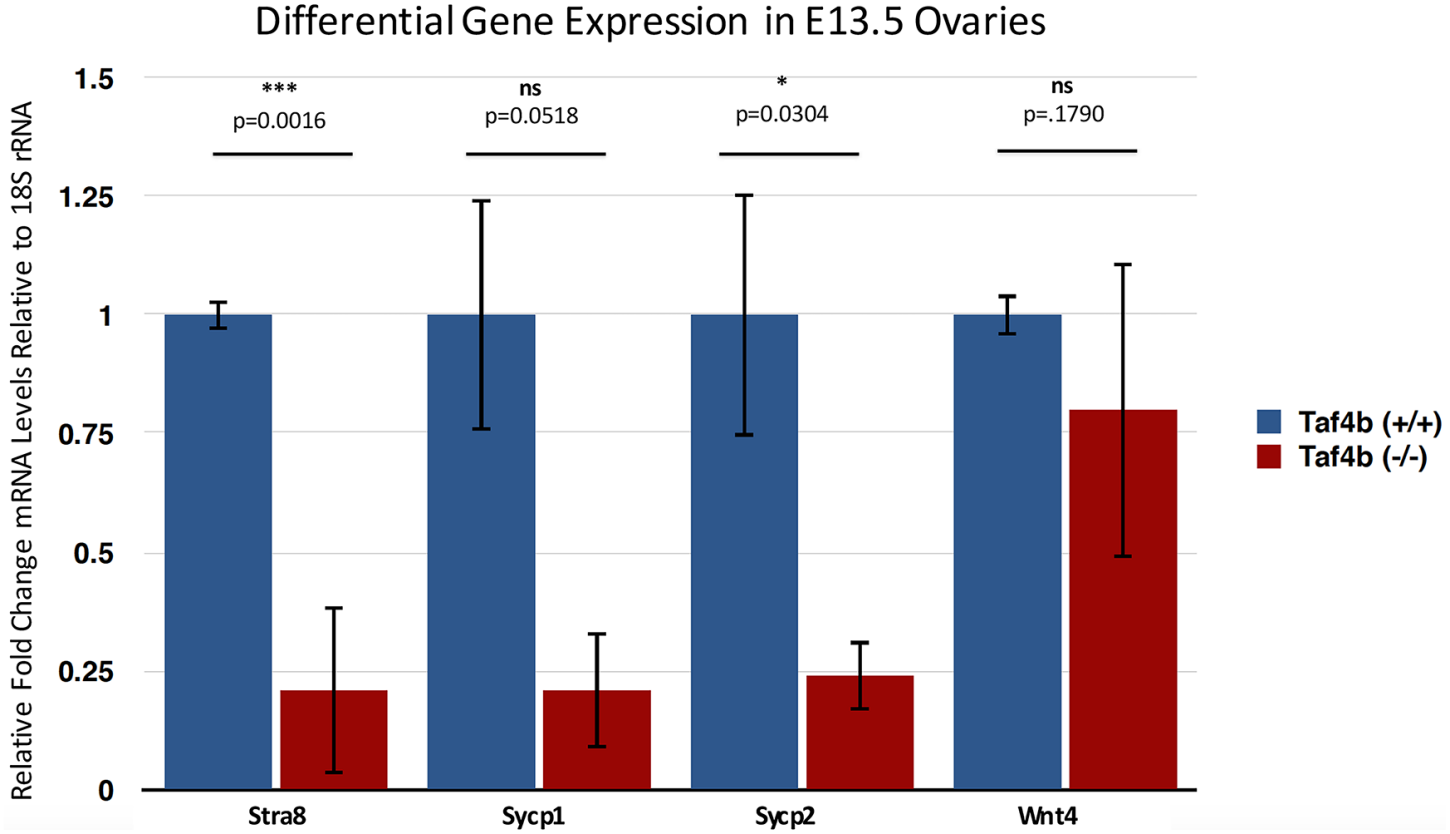
Reduced expression of meiosis genes in E13.5 *Taf4b*-deficient ovaries: Quantitative RT-PCR was used to compare differential gene expression between E13.5 wild-type and *Taf4b*-deficient ovary cDNA. RT-PCR was performed in technical triplicate and values were normalized to 18S rRNA with fold-change displayed after setting the wild-type value to 1. n = 4 wild-type animals and 3 *Taf4b*-deficient animals. *Taf4b*-deficient oocytes displayed about 25% of the *Stra8*, *Sycp1*, and *Sycp2* mRNA expression of wild-type oocytes, while the somatic marker *Wnt4* is not significantly changed between genotypes. Error bars represent SEM, and p-values were determined by Unpaired Student T-Test (* = p < 0.05; *** = p < 0.005).

To better understand if meiotic onset may also be affected in *Taf4b*-deficient oocytes, mRNA expression of *Stra8* was tested at E13.5, the developmental time at which it is first activated by RA [3]. Notably, *Stra8* expression in *Taf4b*-deficient ovaries is approximately 25% of that expressed in wild-type ovaries at the same time (Fig 7). Genes downstream of *Stra8*, including *Sycp1* and *Sycp2*, exhibit similar reduced expression, suggesting that TAF4b may be important for meiotic onset as well as progression through direct transcriptional modulation of *Stra8*. In contrast to these oocyte-specific genes, *Wnt4* expression [36] is not significantly changed in *Taf4b*-deficient ovaries at this time, suggesting that TAF4b is specifically promoting oocyte-specific gene expression programs. Given that oocytes are not mitotic after E13.5, and as we have previously demonstrated equivalent oocyte densities in *Taf4b*-deficient and wild-type ovaries at E18.5 [19], these data likely reflect alterations in gene expression and not reduced germ cell numbers. Accordingly, MVH-stained E13.5 ovaries display relatively equivalent numbers of germ cells across *Taf4b* genotypes (S5 Fig). Proper TAF4b regulation thus leads to the proper expression of downstream genes regulating multiple stages of meiosis I including onset, synapsis, recombination and arrest.

## Discussion

Recent work has converged on the mid- to late-embryonic period in the mouse ovary and mid-gestation development in the human ovary as one of the first critical regulatory periods ensuring the survival of high quality oocytes [4]. Fetal development represents the timeframe in which female germ cells are expanded by mitotic divisions and then dramatically reduced, ultimately producing the apparently finite ovarian reserve. This dynamic proliferation followed by meiotic arrest and significant germ cell apoptosis prior to assembly of primordial follicles coincides with prophase I progression, suggesting that this may represent a quality control mechanism in which only the highest quality oocytes are selected for long-term survival [37]. As such, the regulation of this window of time must not only be finely tuned, but must also result in highly stable oocytes that can maintain genomic integrity and gene expression throughout the reproductive lifespan.

Just prior to the establishment of this finite reserve, faithful progression through prophase I of meiosis ensures genomic integrity by pairing homologous chromosomes, facilitating meiotic crossing over, and ultimately arresting oocytes in a highly stable chromosomal conformation that will persist until ovulation [4]. Disruption of these early meiotic events, as well as altered gene expression, are known causes of accelerated ovarian follicle depletion which can lead to premature reproductive senescence and associated infertility [38]. Here we demonstrate that TAF4b is required in the ovary to properly regulate expression of essential meiosis and oogenesis genes, as well as ensure faithful prophase I progression and recombination, which leads to the proper establishment of the ovarian reserve. These data support a novel role for TAF4b in not only promoting timely initiation of the meiotic program during embryonic development, but also directly occupying the promoters and modulating the expression of genes known to be essential regulators of oogenesis.

ChIP assays revealed a role for TAF4b in directly occupying the promoters of a number of genes that are known “master regulators” of oogenesis including the transcription factors *Figlα* and *Nobox*. Remarkably, *Figlα* and *Nobox* mutant ovaries strongly phenocopy *Taf4b*-deficient ovaries with *Figlα*-deficient mice experiencing germ cell death immediately after birth [12], and *Nobox*-deficient mice exhibiting cyst breakdown defects as well as primordial follicle loss [13,15]. Our studies support a role for TAF4b in regulating the proper expression of these genes, likely through direct transcriptional activation.

Our studies suggest that meiotic errors may underlie the rapid perinatal oocyte loss observed in *Taf4b*-deficient mice. First, we have revealed differential mRNA and protein expression of meiotic effectors including *Sycp1*, *Sycp2*, and MSY2 in *Taf4b*-deficient oocytes. As these effectors all play essential meiotic roles [39–42], we investigated meiotic progression in *Taf4b*-deficient oocytes and observed a lag in the timely progression between the steps of prophase I as well as incomplete synapsis and persistent γH2AX in a majority of *Taf4b*-deficient oocytes. In addition to these striking meiotic errors, the diplotene stage is only reached by a small percentage of *Taf4b*-deficient oocytes at PND0, one day prior to the majority of their excessive attrition.

Furthermore, *Stra8* mRNA expression is approximately 4-fold reduced in E13.5 *Taf4b*-deficient ovaries compared to wild-type, indicating that TAF4b partially controls its expression. As meiotic onset and progression is likely very sensitive to *Stra8* dosage, this diminished *Stra8* expression may underlie the reduced expression of downstream synaptonemal complex protein-encoding genes and the observed meiotic deficits and asynapsis. Interestingly, low levels of *Stra8* expression are evidently sufficient for some degree of meiotic entry. While we are currently unable to discriminate between a prophase I defect as a result of delay in meiotic onset versus other mechanistic errors, the reduction in *Stra8* expression suggests that onset may be affected in *Taf4b*-deficient oocytes. Furthermore, while we cannot exclude altered meiotic onset, our data demonstrating reduced recombination in pachytene *Taf4b*-deficient oocytes suggests that equally-staged oocytes exhibit onset-independent meiotic phenotypes compared to wild-type oocytes. Therefore, we can conclude that in addition to TAF4b playing a role in the timely progression through prophase I, it is also required for other independent and essential meiotic events including homologous recombination.

Interestingly, reduced expression of the RNA-binding protein DAZL may play a key role in the many ovarian phenotypes evident in *Taf4b*-deficient mice. First, DAZL has been shown to bind the 3’ UTR of *Sycp3* transcripts, stabilizing the mRNA and allowing for proper translation and function [43]. Furthermore, *Dazl*-deficient mice also phenocopy *Taf4b*-deficient mice with accelerated germ cell loss around the time of birth. It has also been shown that *Dazl*-deficient mice fail to properly express both *Figlα* and *Nobox* [35], suggesting that in addition to direct occupancy of *Figlα* and *Nobox* promoters, TAF4b may promote their expression and that of *Sycp3* through direct regulation of *Dazl*. Finally, *Dazl* has recently been implicated in the regulation of *Stra8* as well as downstream *Stra8* targets, leading to proper meiotic progression [44]. Therefore, TAF4b-dependent regulation of *Dazl* may help amplify an oocyte-specific gene regulatory network essential for proper meiosis I events.

Our work here agrees with previous research suggesting that meiotic fidelity during the mid-to-late embryonic period is essential for perinatal oocyte survival. For example, other models of meiotic failure, including *Spo11*-deficient mice [45,46] and *Msh4*-deficient mice [47], demonstrate accelerated late embryonic or early postnatal oocyte loss. Indeed, in *Taf4b*-deficient mice as well, we have observed a rapid decline in oocyte density immediately after birth.

**Fig 8:**
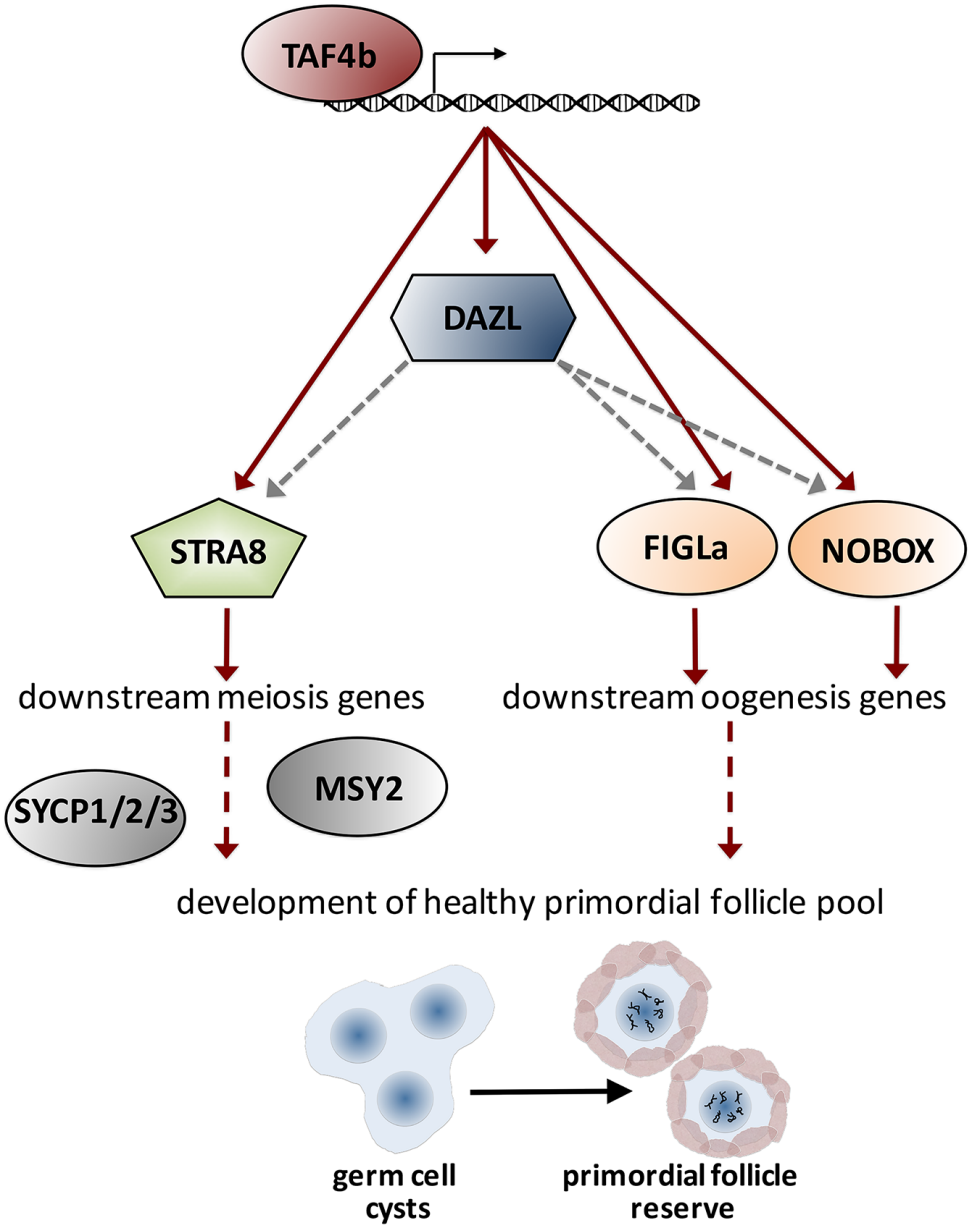
Model of TAF4b promoting a critical meiosis and oogenesis gene regulatory network: Data from chromatin immunoprecipitation experiments demonstrates that TAF4b occupies the proximal promoters of *Dazl*, *Stra8*, *Figlα*, and *Nobox*, ultimately resulting in their expression. Proper expression of these essential regulators facilitates expression of downstream meiosis and oogenesis genes, finally leading to the development of a healthy primordial follicle pool. Red arrows represent direct transcriptional regulation, gray arrows represent post-transcriptional regulation, and dashed lines indicate putative mechanisms.

We propose a model (Fig 8) for TAF4b function in which TAF4b occupies the proximal promoters of a subset of essential meiosis- and oogenesis-regulating genes, including *Stra8*, *Figlα*, *Nobox*, and *Dazl*, distinguishing TAF4b as a novel upstream “regulator of meiotic regulators”. Proper expression of these proteins can then lead to the faithful activation of downstream meiosis and oogenesis genes, including *Sycp1/2/3*, *Msy2*, and *Figlα* and *Nobox* target genes. Ultimately, we propose that TAF4b is required for a gene regulatory network essential for successful prophase I progression and for the establishment of a healthy primordial follicle reserve.

In support of our findings, recent work from Cloutier et al 2015 demonstrates a role for proper meiotic progression and synapsis in perinatal oocyte survival, with asynapsis irrespective of chromosome identity resulting in diplonema oocyte loss [48], as similarly observed in *Taf4b*-deficient mice. These authors suggest that oocyte death results from reduced expression of essential oogenesis genes positioned on asynapsed chromosomes due to persistence of γH2AX in pachytene, as seen in *Taf4b*-deficient oocytes. As we have demonstrated a role for TAF4b in the proper expression of a number of meiotic regulators including synaptonemal complex genes, our data suggests that reduced expression of these factors results in an inability to properly synapse, γH2AX-dependent meiotic silencing, and diplotene oocyte loss. These data further highlight the fine-tuning of transcription that must be achieved for healthy oogenesis and folliculogenesis.

We propose that TAF4b regulates this process by two potentially independent mechanisms: first, directly occupying and promoting transcription of oogenesis genes, and second, playing a role in the timely and healthy progression through prophase I. Remarkably, human TAF4B may play an analogous role to mouse TAF4b in the establishment of a woman’s ovarian reserve while she is still developing *in utero*. As we have demonstrated coordinate regulation of human *TAF4B* and human *SYCP3*, *YBX2*, and *DAZL* expression (along with that of many other meiosis genes), TAF4B may possess a conserved function in regulating meiotic progression and oocyte survival in both mice and women.

## Materials and Methods

### Animals

Wild-type (*Taf4b* (+/+)) and *Taf4b*-deficient (-/-) or heterozygous (+/-) mice were generated by mating heterozygous *Taf4b* (+/−) male and female mice as described previously [16]. Offspring were genotyped by PCR analysis of tail-snip genomic DNA amplifying the region targeted by homologous recombination. All animal protocols were reviewed and approved by Brown University Institutional Animal Care and Use Committee and were performed in accordance with the National Institutes of Health Guide for the Care and Use of Laboratory Animals (# 1503000130).

### Ovarian immunofluorescence

Ovaries were removed, cleaned of excess fat, and fixed in 4% formaldehyde solution overnight before embedding in Optimal Cutting Temperature (OCT) Compound. Ovaries were serially sectioned at 8 μm on a Leica Cryostat onto glass slides and washed in 1x Phosphate-Buffered Saline (PBS) containing 0.1% Triton-X (CalBiochem). The entire tissue was sectioned and the median, adjacent two sections chosen for staining and quantification. Tissue sections were then incubated in blocking buffer [3% Goat Serum (Sigma), 1% Bovine Serum Albumin (Sigma), and 0.5% Tween-20 (Fisher Scientific) in 1X PBS] and stained by incubation with primary antibodies against TRA98 and MSY2 (Abcam) for diplotene analysis, primary antibody against DAZL (Abcam) for expression analysis, or primary antibody against Mouse Vasa Homolog (Abcam) for E13.5 germ cell analysis. A secondary antibody-only control was included to compare background staining. Sections were further stained with DAPI to visualize nuclei and analyzed on an Epifluorescent Zeiss Axioplan microscope.

Percentages of MSY2-positive oocytes were determined by counting cells positive for both TRA98 and MSY2 and dividing by the total number of TRA98-positive cells per section. Results were averaged and significance determined by two-tailed unpaired t-test. Error bars represent standard error of the mean.

### Meiotic prophase spreads

Chromosome spreads were prepared by the “drying down technique” as previously described [49]. Briefly, ovaries were collected from E16.5 mice and incubated in warmed PBS until their use. Ovaries were then incubated in hypotonic extraction buffer [30 mM Tris, 50 mM sucrose, 17 mM trisodium citrate dihydrate, 5 mM EDTA, 0.5 mM DTT, and 0.5 mM phenylmethylsulphonyl fluoride (PMSF), pH 8.2] for 30 minutes, then teased apart in 100 mM sucrose. The ovarian single cell suspension was then pipetted onto slides wetted in 1% PFA with 0.2% Triton X-100 and allowed to settle overnight in a humid chamber at 37°C. The next day, slides were air-dried, incubated in 0.4% Photo-Flo (Kodak) for 2 minutes, air-dried again and then either stained or stored at -80°C. Spreads were stained by immunofluorescence as described above using primary antibodies against MLH1 (BD Pharmigen), SYCP1 (Abcam), CENP-A (Abcam), γH2AX (Millipore) or SYCP3 (Santa Cruz). To determine prophase I stage, SYCP3 configuration (as shown in S1 Fig) was used. To determine percent oocyte asynapsis, >20 CENP-A centromeric foci was used as a quantitative assessment. For both analyses, four animals yielding approximately 50 oocytes and 10–20 pachytene oocytes each were utilized. To quantify meiotic recombination, 25 or more pachytene oocytes per animal were scored for MLH1 foci on chromosome cores as visualized by SYCP3 staining.

### TAF4b-specific antibody production and purification

The N-terminal TAF4b amino acids 1–255 were fused to a 6xHis tag by PCR amplification and subcloned into the pETRB-1P expression vector (a generous gift from Dr. Rebecca Page, Brown University). The 6xHis-TAF4b recombinant protein was expressed in *E*. *coli* BL21(DE3) Rosetta cells (EMD Millipore, Billerica, MA) and the cell pellet was resuspended in protein purification buffer (8M Urea, 100 mM NaCl, 20 mM HEPES pH 8.0 and Complete Protease Inhibitor Cocktail (Roche, Indianapolis, IN)). The cells were lysed by freeze-thawing at -80°C and 37°C 3 times, sonicated and insoluble material was removed by centrifugation. The 6xHis-TAF4b protein was purified from the soluble protein lysate using Ni-agarose affinity chromatography and elution with imidazole. Protein purification was assayed by SDS-PAGE and Coomassie staining (S4A Fig). Recombinant 6xHis-TAF4b were concentrated to 1–2 mg/ml by dialysis, lyophilization and resolubilization in 6M Urea. Polyclonal antibodies against mouse TAF4b were then generated by immunizing rabbits and chickens (Cocalico Biologicals Inc., Reamstown, PA). Recombinant 6xHis-TAF4b protein was crosslinked to AminoLink Coupling Resin (Life Technologies, Grand Island, NY) and the polyclonal antibodies were affinity-purified from both the rabbit and chicken antisera (S4 Fig). Antibody specificity was assayed by immunoblot analysis following antibody pre-incubation with recombinant 6xHis-TAF4b protein and immunoprecipitation capability was tested (S4B–S4E Fig).

### Chromatin immunoprecipitation

ChIP from wild type E18.5 ovaries was performed on a pooled sample of ten ovaries from five embryos. Tissue was cross-linked using 1.5% formaldehyde for 20 minutes at room temperature, then quenched with 0.125 M glycine for 5 minutes. Tissue was then washed twice in ice-cold PBS and dounced into a single cell suspension in PBS with 1 mM PMSF and 1X Complete Mini Protease Inhibitor (Roche). Dissociated tissue was then spun and resuspended in ChIP Lysis Buffer (1% SDS, 10 mM EDTA, and 50 mM Tris, pH 8.1). Chromatin was sheared into 200-1000bp fragments using a Covaris sonicator. Resulting chromatin was spun, the supernatant saved, and used for the remainder of the procedure.

Chromatin was pre-cleared using Protein A Agarose beads (GE Healthcare) in ChIP Dilution Buffer (0.01% SDS, 1.1% Triton X-100, 1.2 mM EDTA, 16.7 mM Tris-HCl pH 8.1, and 167 mM NaCl), rotating for one hour. Immunoprecipitation was then performed using 20 μg of affinity-purified anti-rabbit TAF4b antibody or 20 μg of anti-IgG antibody (Santa Cruz), rotating overnight. A third sample was rotated overnight with no primary antibody for a beads-only control. The next day, Protein A Agarose beads were added back to the sample, rotating for one hour before spinning and collecting the beads. Protein-DNA complexes were then washed in sequence with Low Salt Immune Complex Wash Buffer (0.1% SDS, 1% Triton X-100, 2 mM EDTA, 20 mM Tris-HCl pH 8.1, and 150 mM NaCl), High Salt Immune Complex Wash Buffer (0.1% SDS, 1% Triton X-100, 2 mM EDTA, 20 mM Tris-HCl pH 8.1, and 500 mM NaCl), LiCl Immune Complex Wash Buffer (0.25 M LiCL, 1% IGEPAL CA630, 1% deoxycholic acid, 1 mM EDTA, and 10 mM Tris-HCl pH 8.1), and TE Buffer (10 mM Tris-HCl pH 8.1 and 1 mM EDTA). Protein-DNA complexes were eluted from the beads by incubating in Elution Buffer (1% SDS and 100 mM NaHCO_3_) for 30 minutes and collecting the supernatant. DNA was purified by reversing crosslinks overnight at 65°C with the addition of 5 M NaCl, treatment with RNase A for 30 minutes at 37°C, and Proteinase K for 1 hour at 45°C. DNA was collected by binding to and eluting from Qiagen DNA-binding spin columns.

Purified DNA was then analyzed by standard PCR amplification of specific regions of candidate TAF4b target promoters: *Stra8*, *Dazl*, *Figlα*, *Nobox*, *Sycp2*, *Sycp3*, and *Msy2*. Purified DNA was also analyzed by PCR amplifications of regions that are not considered candidate TAF4b target regions including a non-genic region 50 kb upstream of the *Nobox* promoter. Primer sets were designed to amplify 100–175 base pair-sized products (S2 Table). The ABI 7900H Real-Time PCR system (Applied Biosystems) and the Power SYBR Green qPCR Master Mix with ROX (Invitrogen, Carlsbad, CA) were used for qPCR data acquisition. Data from each primer set were normalized to the E18.5 mouse ovary genomic DNA input levels and represented as a percentage of that DNA input. Each qPCR reaction was performed in triplicate and averaged. Error bars indicate the normalized standard deviation resulting from experimental triplicate qPCR reactions.

Proximal promoter regions were identified using the GRCm38.p3 *Mus musculus* genomic assembly and performing a chromosome coordinate sequence download from NCBI. Sequences 0 to 500 base pairs upstream of the NCBI reported gene contigs were called as proximal promoters. All sequences were then independently confirmed for genomic position using UCSC Genome Browser.

### Quantitative real-time reverse transcription PCR

Whole ovaries were collected from E13.5 *Taf4b*-wild-type and -deficient embryos and total RNA was extracted by Trizol extraction and lithium chloride precipitation. Total RNA from all experiments was quantified and checked for purity, and 50 ng was used to prepare 20 μl of cDNA with an iScript cDNA Synthesis Kit (Bio-Rad). Real-time PCR was performed in technical triplicate using 1 μl of DNA template, 10 μl of ABI SYBR green PCR master-mix (Applied Biosystems), and 0.5 μM custom oligos (Invitrogen) for *Stra8*, *Sycp1*, *Sycp2*, *Sycp3*, *Wnt4*, or *18S* rRNA in a 20 μl reaction in an ViiA 7 Real Time PCR machine (Life Technologies). Data were analyzed by the ΔΔCt method, and relative expression levels were normalized to *18S* rRNA. Error bars represent the standard deviation of the fold-change over wild-type of normalized gene expression values. P values were determined by two-tailed unpaired Student t-test. Primer sequences corresponding to genes of interest can be found in S3 Table.

### Correlation analysis

Gene expression from human ovaries was analyzed in the GEO dataset, GSE15431. Pearson correlation coefficients and P-values were calculated in Microsoft Excel. Pathway enrichments were determined using Ingenuity Pathway Analysis (IPA) (Qiagen).

## Supporting Information

S1 Fig Synaptonemal Complex Protein 3 configuration during prophase I: Wild-type oocyte meiotic chromosome spreads were prepared and stained with primary antibodies against Synaptonemal Complex Protein 3; configuration of SYCP3 was used to determine prophase I stage, as indicated by the four panels. (TIFF)

S2 Fig Recombinant TAF4b protein purification, polyclonal anti-TAF4b antibody production and antibody purification. (A) Coomassie-stained SDS-PAGE analysis of 6xHis-TAF4bN1 recombinant protein purification. Immunoblot detection of recombinant 6xHis-TAF4bN1 antigen with preimmune and anti-sera from chicken (B) and rabbit (C) (predicted molecular weight of 28kDa). Antigen-specific competition of endogenous TAF4b protein immunoblot detection using rabbit (D) and chicken (E) affinity-purified antibodies. (F) Specific immunoprecipitation of endogenous P46 mouse testis extracts with affinity-purified rabbit anti-TAF4b antibodies and immunoblot detection with affinity-purified chicken anti-TAF4b antibodies. All arrowheads indicate the 105kDa full-length form of TAF4b.
(TIFF)

S3 Fig TAF4b targets the promoters of critical meiosis and oogenesis regulators: Wild-type E18.5 ovarian chromatin pulled down by antibodies against TAF4b or IgG and then PCR-amplified using primers against the proximal promoters of *Stra8*, *Dazl*, *Figlα*, *Nobox*, and a non-genic region upstream of *Nobox*. “Input” lanes indicate the positive control PCR reaction, while “beads alone” and “no template” lanes indicate negative control PCR reactions. *:non-specific priming bands.
(TIFF)

S4 Fig Reduced DAZL protein expression in PND0 *Taf4b*-deficient ovaries. (A) PND0 wild-type and *Taf4b*-deficient (B) ovary tissue sections were stained with antibodies against DAZL and TRA98, showing reduced DAZL expression in *Taf4b*-deficient ovaries. (C) Western blot analysis was used to confirm the immunostaining results, with the blot incubated in primary antibodies against DAZL and beta-tubulin as a loading control.
(TIFF)

S5 Fig E13.5 wild-type and *Taf4b*-deficient ovary tissue sections were stained with primary antibody against MVH to visualize germ cells at the start of female meiosis. Both genotypes possess relatively similar numbers of germ cells at this embryonic time.
(TIFF)

S1 Table Ingenuity pathway analysis of *TAF4B*-correlated genes. Coordinate gene expression profiles of human fetal ovary over gestational time were analyzed with Pearson Correlations and Ingenuity Pathway Analysis. Human *TAF4B* was found to be most highly correlated with the expression of a network of meiotic regulators.
(XLSX)

S2 Table Chromatin immunoprecipitation primers. Primers used for amplification of chromatin immunoprecipitated from E18.5 fetal mouse ovary are listed here. Primers amplified a 100–200 base pair fragment at the genomic region indicated.
(DOCX)

S3 Table Quantitative RT-PCR primers. Primers used for amplification of cDNA from E13.5 fetal mouse ovary are listed here. Primers amplified a 100–200 base pair fragment of the gene indicated.
(DOCX)
