# Supplementary figures and images for: TAF4b Regulates Oocyte-Specific Genes Essential for Meiosis
Source: PLoS Genet. 2016 Jun 24;12(6):e1006128. doi: 10.1371/journal.pgen.1006128 (PMC4920394; doi:10.1371/journal.pgen.1006128)

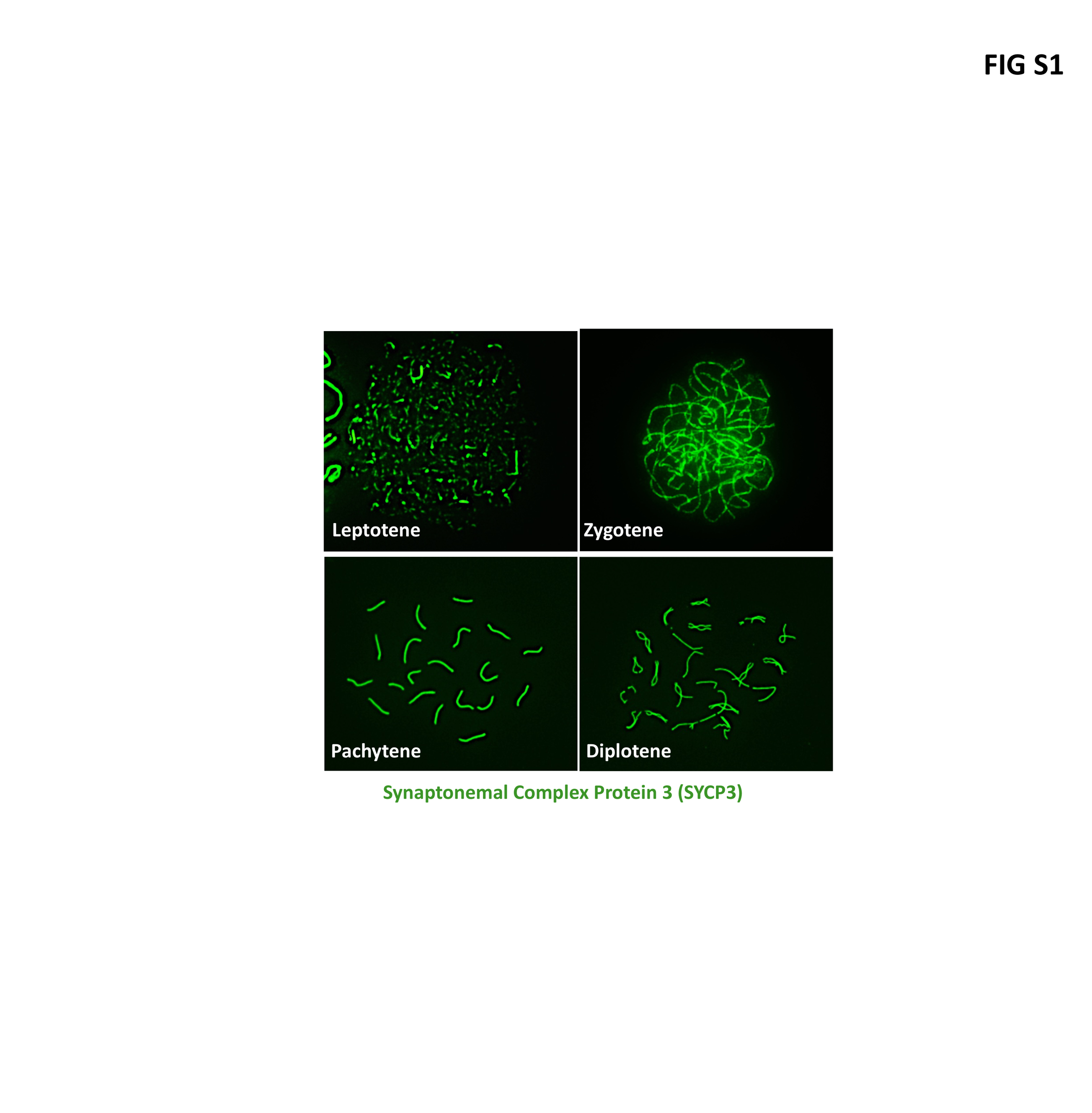

Supplement: S1 Fig — (TIFF) [file pgen.1006128.s001.tiff]

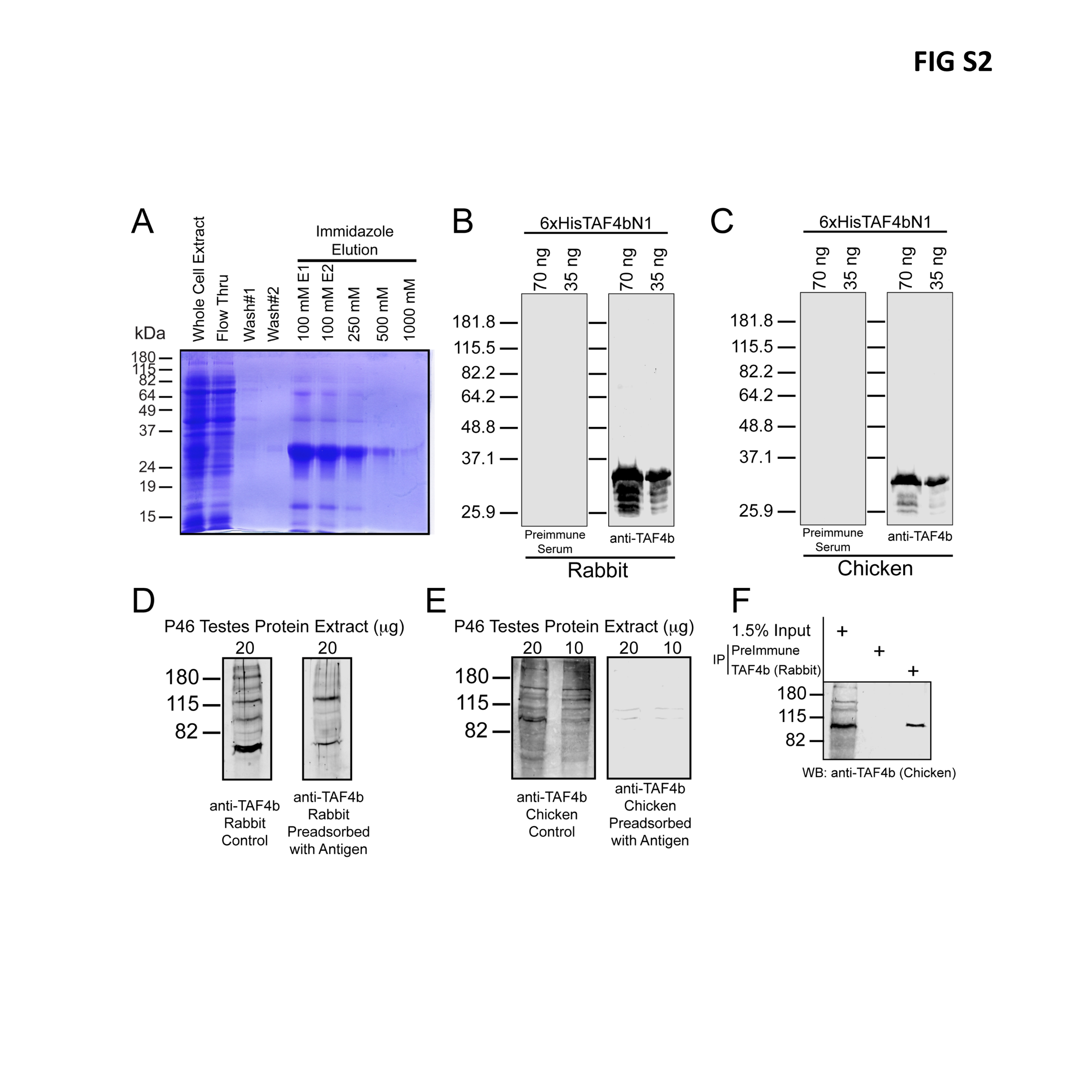

Supplement: S2 Fig — (A) Coomassie-stained SDS-PAGE analysis of 6xHis-TAF4bN1 recombinant protein purification. Immunoblot detection of recombinant 6xHis-TAF4bN1 antigen with preimmune and anti-sera from chicken (B) and rabbit (C) (predicted molecular weight of 28kDa). Antigen-specific competition of endogenous TAF4b protein immunoblot detection using rabbit (D) and chicken (E) affinity-purified antibodies. (F) Specific immunoprecipitation of endogenous P46 mouse testis extracts with affinity-purified rabbit anti-TAF4b antibodies and immunoblot detection with affinity-purified chicken anti-TAF4b antibodies. All arrowheads indicate the 105kDa full-length form of TAF4b. (TIFF) [file pgen.1006128.s002.tiff]

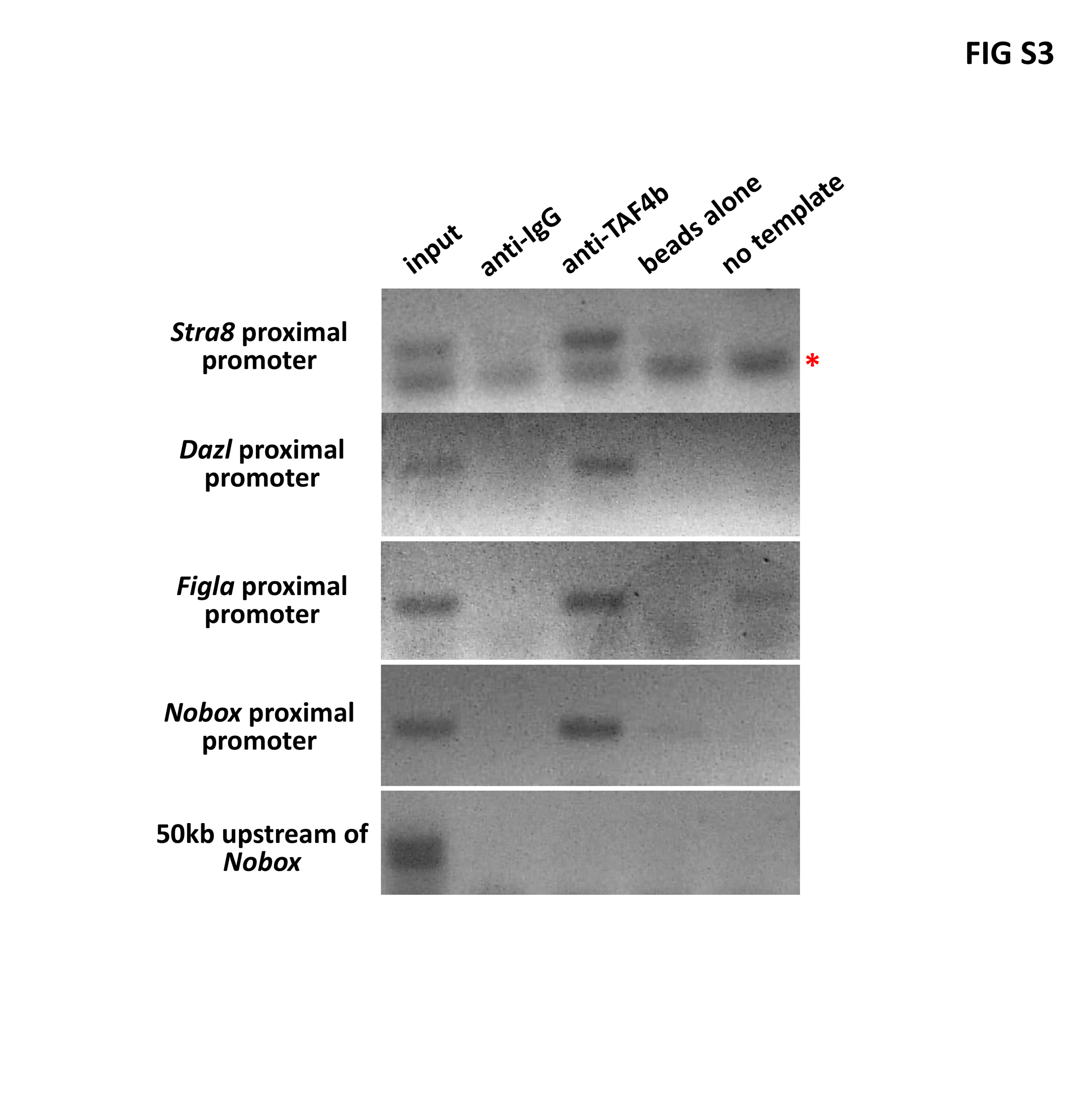

Supplement: S3 Fig — “Input” lanes indicate the positive control PCR reaction, while “beads alone” and “no template” lanes indicate negative control PCR reactions. *:non-specific priming bands. (TIFF) [file pgen.1006128.s003.tiff]

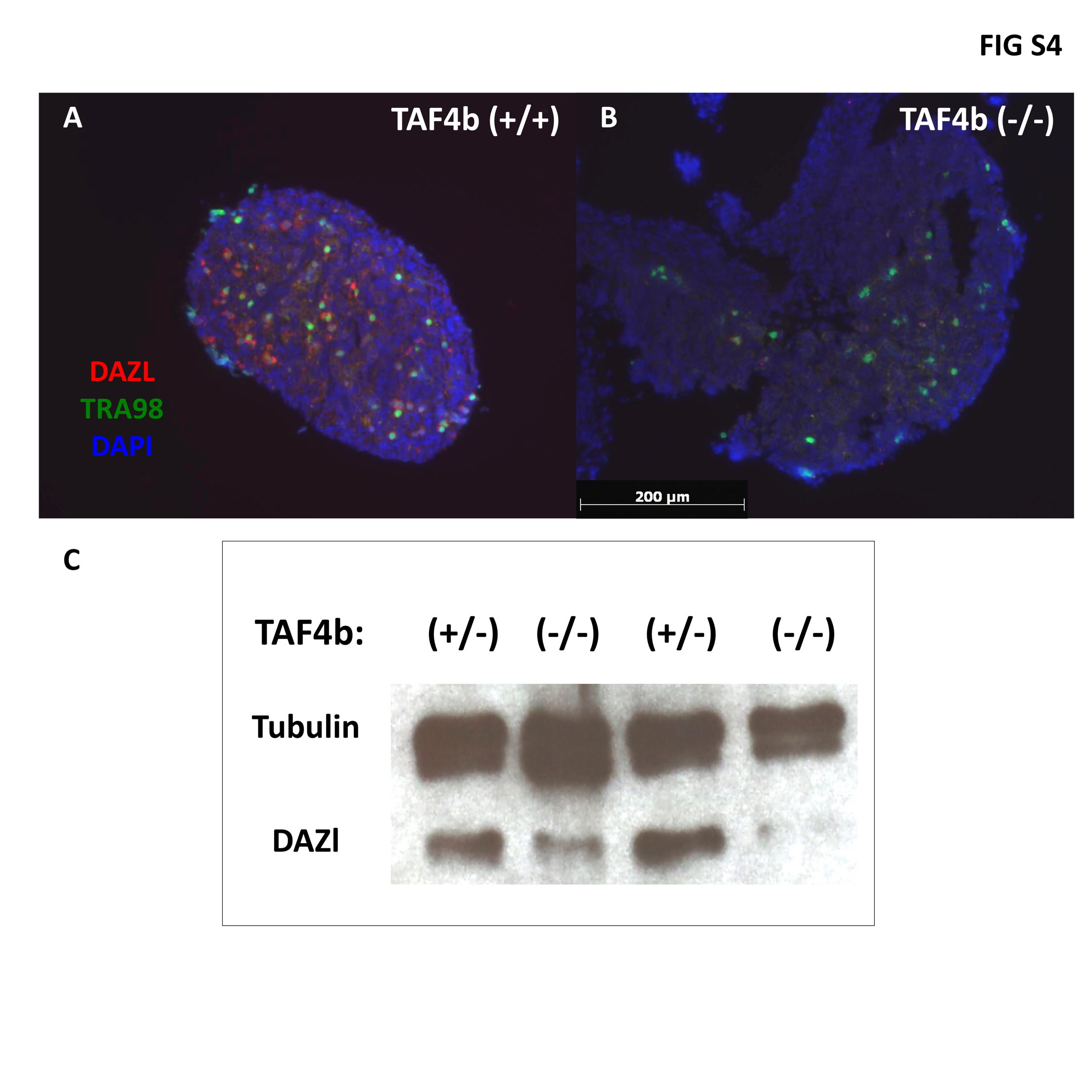

Supplement: S4 Fig — (A) PND0 wild-type and Taf4b-deficient (B) ovary tissue sections were stained with antibodies against DAZL and TRA98, showing reduced DAZL expression in Taf4b-deficient ovaries. (C) Western blot analysis was used to confirm the immunostaining results, with the blot incubated in primary antibodies against DAZL and beta-tubulin as a loading control. (TIFF) [file pgen.1006128.s004.tiff]

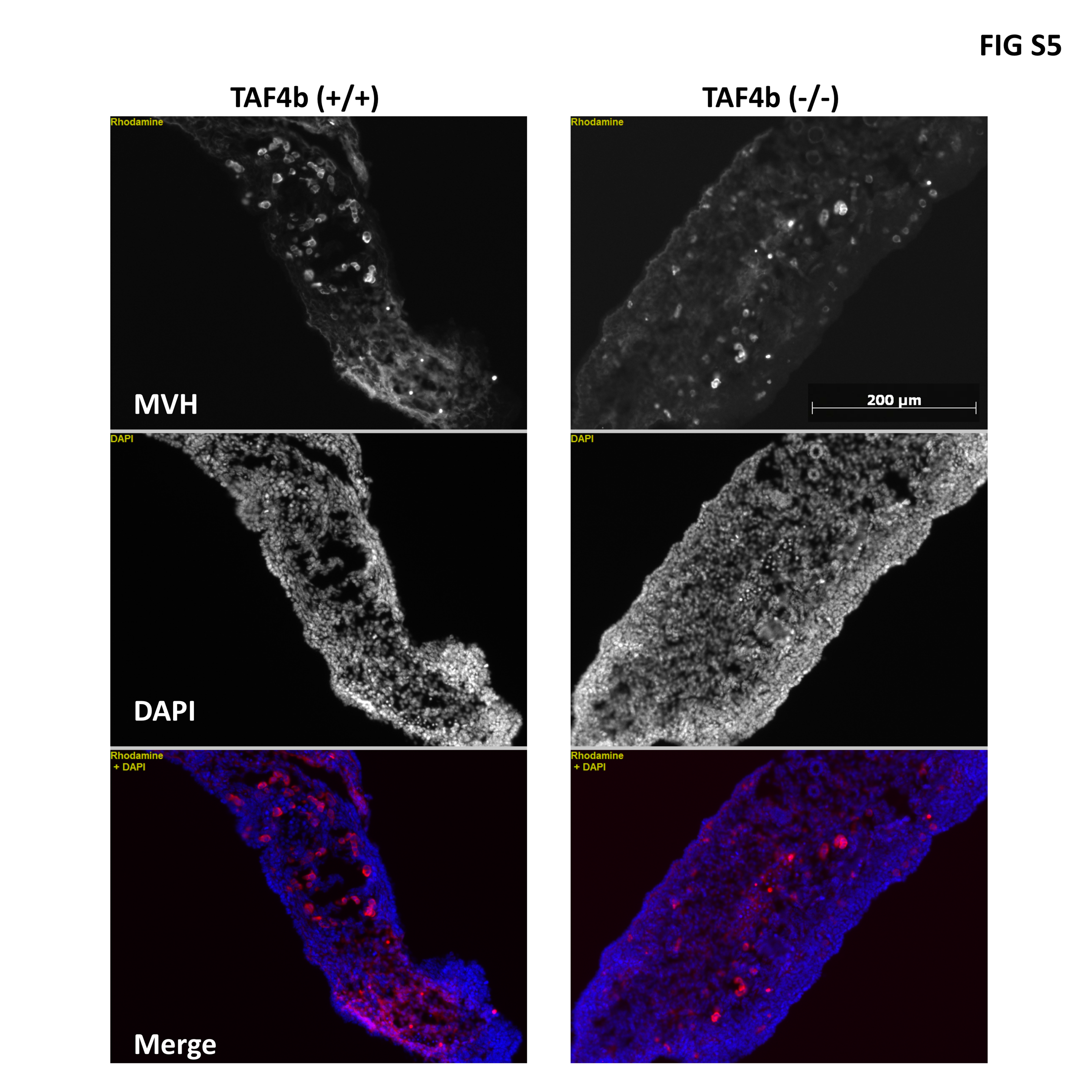

Supplement: S5 Fig — Both genotypes possess relatively similar numbers of germ cells at this embryonic time. (TIFF) [file pgen.1006128.s005.tiff]
