# Supplementary material for: TAF4b Regulates Oocyte-Specific Genes Essential for Meiosis
Source: PLoS Genet. 2016 Jun 24;12(6):e1006128. doi: 10.1371/journal.pgen.1006128 (PMC4920394; doi:10.1371/journal.pgen.1006128)
Supplement: S2 Table — Primers used for amplification of chromatin immunoprecipitated from E18.5 fetal mouse ovary are listed here. Primers amplified a 100–200 base pair fragment at the genomic region indicated. (DOCX) [file pgen.1006128.s007.docx]

Table S2 - ChIP PCR and qPCR Primers

| **Gene** | **Location Relative to Transcription Start Site** | **Primer Sequence** |
| --- | --- | --- |
| *Stra8* | 50bp upstream | Forward - GTC CCT ACC ATT CAG GAG CA |
|  |  | Reverse - ATG CGT GCT TCC TTC CAT AC |
| *Dazl* | 50bp upstream | Forward - AAG CTT TGC TGA CGG GAT AA |
|  |  | Reverse - CAG ACA GAT GGA CCG ACT |
| *Figla* | 50bp upstream | Forward - TCC TCC TCT TAC CCT CTC AGG |
|  |  | Reverse - ATG CGC AAG GAG CTT AAC TG |
| *Nobox* | 50bp upstream | Forward - CCT TCC ATT GCT GTT CTG CT |
|  |  | Reverse - CCA GGT GTG TGG AGT CTG C |
| *Nobox* | 50,000bp (50kb) upstream | Forward - TCA GTG AGC AAT GTC TCT AAG CA |
|  |  | Reverse - TGC ACT GAA GTG TCA GAT TCA C |
