# Supplementary material for: TAF4b Regulates Oocyte-Specific Genes Essential for Meiosis
Source: PLoS Genet. 2016 Jun 24;12(6):e1006128. doi: 10.1371/journal.pgen.1006128 (PMC4920394; doi:10.1371/journal.pgen.1006128)
Supplement: S3 Table — Primers used for amplification of cDNA from E13.5 fetal mouse ovary are listed here. Primers amplified a 100–200 base pair fragment of the gene indicated. (DOCX) [file pgen.1006128.s008.docx]

Table S3 – E13.5 qRT-PCR Primers

| **Gene** | **Primer Sequence** |
| --- | --- |
| *Stra8* | Forward - ACCCTGGTAGGGCTCTTCAA |
|  | Reverse - GACCTCCTCTAAGCTGTTGGG |
| *Scp1* | Forward - CAAAAGCCCTTCACACTGTTCG |
|  | Reverse - GTTTTCCCGACTGGACATTGTAA |
| *Scp2* | Forward – AGGATGAGATCACTACACCTAGC |
|  | Reverse – GGTGACGCAGCATAATCCATT |
| *Wnt4* | Forward - AAG AGG AGA CGT GCG AGA AAC |
|  | Reverse - GTC CCT TGT GTC ACC ACC TT |
| *18S* rRNA | Forward – GTAACCCGTTGAACCCCATT |
|  | Reverse – CCATCCAATCGGTAGTAGCG |
